# Vitamin D supplementation in the management of knee osteoarthritis: study protocol for a randomized controlled trial

**DOI:** 10.1186/1745-6215-13-131

**Published:** 2012-08-06

**Authors:** Yuelong Cao, Graeme Jones, Flavia Cicuttini, Tania Winzenberg, Anita Wluka, James Sharman, Kay Nguo, Changhai Ding

**Affiliations:** 1 Menzies Research Institute Tasmania, University of Tasmania, Private Bag 23, Hobart, TAS, 7000, Australia; 2 Department of Epidemiology and Preventive Medicine, Monash University, Melbourne, 3004, Australia; 3 Research Institute of Orthopaedics, Shuguang Hospital Affiliated to Shanghai University of Traditional Chinese Medicine, Shanghai, 201203, China

**Keywords:** Vitamin D, Osteoarthritis, Magnetic resonance imaging

## Abstract

**Background:**

Osteoarthritis (OA) is a common health issue worldwide in the aging population who are also commonly deficient in vitamin D. Our previous study suggested that higher serum 25-(OH)D levels were associated with reduced knee cartilage loss, implying that vitamin D supplementation may prevent the progression of knee OA. The aim of the VItamin D Effects on OA (VIDEO) study is to compare, over a 2- year period, the effects of vitamin D supplementation versus placebo on knee structural changes, knee pain, and lower limb muscle strength in patients with symptomatic knee OA.

**Methods/design:**

Randomised, placebo-controlled, and double-blind clinical trial aiming to recruit 400 subjects (200 from Tasmania and 200 from Victoria) with both symptomatic knee OA and vitamin D deficiency (serum [25-(OH)D] level of >12.5 nmol/liter and <60 nmol/liter). Participants will be randomly allocated to vitamin D supplementation (50,000 IU compounded vitamin D_3_ capsule monthly) or identical inert placebo group for 2 years. The primary endpoint is loss of knee cartilage volume measured by magnetic resonance imaging (MRI) and Western Ontario and McMaster Universities Index of OA (WOMAC) knee pain score. The secondary endpoints will be other knee structural changes, and lower limb muscle strength. Several other outcome measures including core muscle images and central blood pressure will be recorded. Linear and logistic regression will be used to compare changes between groups using univariable and multivariable modeling analyses. Both intention to treat and per protocol analyses will be utilized.

**Discussion:**

The trial is designed to test if vitamin D supplementation will reduce loss of knee cartilage volume, prevent the progression of other knee structural abnormalities, reduce knee pain and strengthen lower limb muscle strength, thus modify disease progression in knee OA.

**Trial registration:**

ClinicalTrials.gov identifier: NCT01176344; Australian New Zealand Clinical Trials Registry: ACTRN12610000495022

## Background

Osteoarthritis (OA) is the most common form of arthritis in the world and one of the most common chronic conditions managed in Australian general practice [1,2]. It is characterized by the gradual loss of articular cartilage and changes to other joint structures eventually leading to total joint replacement. Currently there is no cure for OA, and the development of innovative and cost-effective approaches to prevent the development and progression of OA is urgent and important.

Vitamin D comprises a group of fat-soluble secosteroids emcompassing two major molecules, vitamin D_2_ and vitamin D_3._ Vitamin D is circulated to the liver where it is converted to the prohormone calcidiol, or 25-hydroxy-vitamin D (25-(OH)D), which is the best indicator of vitamin D status [3-5]. Vitamin D deficiency is very common in older people. It is estimated that 20 to 100% of elderly men and women in North America and Europe are vitamin D deficient (mostly defined as a serum level of 25-(OH)D < 50 nmol/liter) [6,7]. High rates of vitamin D deficiency have also been reported in all sectors of the community of Australia, especially in Tasmania and Victoria where this study will be conducted [8-10].

It has been widely recognized that OA is a disease affecting the whole joint, including cartilage, bone and muscle. Through targeting these joint tissues vitamin D supplementation may modify disease progression in OA. Vitamin D receptors (VDRs) are found in human articular chondrocytes [11], and 1α-25(OH)_2_D_3_ regulates the expression of metalloproteinase (MMP) and prostaglandin E_2_ (PGE_2_) in chondrocytes via VDRs [11]. Vitamin D could enhance the ability of bone to respond optimally to pathophysiological processes in OA, thus prevent disease progression [12,13]. Furthermore, 1,25(OH)_2_D leads to *de novo* protein synthesis, muscle cell growth, and improved muscle function, and thus has a beneficial effect on muscle strength [14].

Epidemiological studies have provided preliminary evidence supporting the potential use of vitamin D for the treatment of OA. Lower serum levels of 25-(OH)D were associated with greater knee pain [15] and higher prevalence of radiographic OA [16], and predicted incidence of knee pain [17], progression/incidence of radiographic OA [18,19], and loss of joint space, as well as osteophyte growth [18]. Magnetic resonance imaging (MRI) has been utilized to directly assess knee structural alterations such as cartilage volume, cartilage defects, subchondral bone changes and meniscal lesions. Using MRI, we reported that, in cross-sectional analysis, serum 25-(OH)D level was significantly and positively associated with knee cartilage volume in older men and women, and vitamin D insufficiency was positively associated with medial and lateral tibial bone area in women. Longitudinally, higher baseline serum levels of 25-(OH)D predicted reduced loss of cartilage volume over 2 years, and increases in vitamin D levels were associated with further protective association [15,16]. Furthermore, serum levels of 25-(OH)D were also associated with increased leg muscle strength and quality, and thus may be important for the maintenance of muscle function [20].

Based on this experimental and epidemiological evidence, we have initiated a randomized, placebo-controlled trial (Vitamin D Effect on Osteoarthritis, VIDEO study) to determine if vitamin D supplementation can reduce loss of knee cartilage volume, prevent the progression of other knee structural abnormalities and strengthen lower limb muscle strength, and thus modify disease progression in knee OA. The effects of vitamin D supplementation on the progression of knee pain will also be determined.

In a sub-study, we will examine the effects of vitamin D supplementation on the function of deep lumbo-pelvic stabilising muscles. The protective deep lumbo-pelvic stabilising muscles, which include (but not exclusively) the transversus abdominus (TrAb) and lumbar multifidus and which are known as core muscles, become dysfunctional shortly after the onset of low back pain, and that ongoing muscle dysfunction is associated with persistent low back pain [18,21]. Muscle weakness can be a sign of vitamin D deficiency [19] and therefore, vitamin D supplementation may have beneficial effects on functionally important core muscles.

Furthermore, we will determine the effect of vitamin D supplementation on blood pressure (clinical, ambulatory, upper arm and central measures) and arterial stiffness (aortic pulse wave velocity). Several lines of evidence suggest that vitamin D deficiency may influence blood pressure via mechanisms including activation of the renin-angiotensin-aldosterone system (RAAS) [22-24]. Central and ambulatory blood pressure will be the main outcomes because central blood pressure is the actual pressure load experienced by the heart (and other organs such as the kidneys and brain) rather than the pressure at the upper arm [25], and ambulatory blood pressure is regarded as the gold standard technique because it correlates with target organ damage and provides more accurate information on daily (including night time) blood pressure fluctuations [26].

## Methods/design

### Study design

VIDEO is a randomized, placebo-controlled double-blind clinical trial. Four hundred subjects (200 from Tasmania and 200 from Victoria) with symptomatic knee OA and serum 25-(OH)D > 12.5 nmol/liter and < 60 nmol/liter will be recruited and randomly allocated to either the treatment or placebo control group. Recruitment methods will include advertisements through the local media and community groups as well as liaisons with general practitioners, specialist rheumatologists, and orthopedic surgeons. Ethics approval has been received from The Tasmania Health and Human Medical Research Ethics Committee (reference number H1040) and Monash University Human Research Ethics Committee (reference number CF10/1182 - 2010000616). Informed written consent will be obtained from all participants.

### Inclusion criteria

The inclusion criteria were as follows: age 50 to 79 years; symptomatic knee OA for at least 6 months with a pain at least 20 mm on a 100 mm visual analogue scale (VAS); American College of Rheumatology (ACR) criteria for symptomatic knee OA assessed by a rheumatologist [27]; ACR functional class rating of I, II and III [28]; relatively good health, with a score of 0 to 2 on a 5-point Likert scale (with a range of 0 indicating very good health to 4 indicating very poor health), according to the investigators global assessment of disease status; serum 25-(OHD)D > 12.5 nmol/liter and < 60 nmol/liter; able to read, speak and understand English, capable of understanding the study requirements and willing to cooperate with the study instructions.

### Exclusion criteria

Exclusion criteria were as follows: severe radiographic knee OA, grade 3 according to Altman’s atlas [29]; severe knee pain on standing (more than 80 mm on 100-mm VAS); any contraindication to having MRI; rheumatoid or psoriatic arthritis, lupus or cancer; severe cardiac or renal impairment; hypersensitivity to vitamin D; any condition possibly affecting oral drug absorption (for example, gastrectomy or malabsorption syndromes); significant trauma to knees, including arthroscopy or significant injury to ligaments or menisci of the knee within one preceding the study; anticipated need for knee or hip surgery within the next 2 years; history of taking vitamin D supplements within the previous 30 days; history of taking an investigational drug within the previous 30 days.

### Randomization

Participants in each site will be randomly assigned to the intervention arm or placebo arm in a ratio of 1:1 and the randomization will be double-blind. Allocation of participants will be based on computer-generated random numbers. Allocation concealment will be ensured by the use of an identical inert placebo, and a central automated allocation procedure, with security in place to ensure allocation data cannot be accessed or influenced by any person.

### Intervention

Participants in the intervention arm will take one capsule per month of 50,000 IU (1.25 mg) of a vitamin D3 compound (cholecalciferol), purchased from Nationwide Compounding Pharmacy, Melbourne, Australia [30], and patients in the control arm will receive an identical inert placebo provided by the same company. Patients are required to record their medication information in personal diaries and a reminder will be given each month. All participants will receive the recommended standard of care. The duration of the study is 2 years.

### Quality assurance

To ensure that this trial will be of a high standard and delivered in accordance with the trial protocol, all research staff will be provided with a standard protocol and case report form, and will be trained to competently administer items as per protocol. The investigators, research assistants and outcome assessors are different people. Protocols will not be altered during the study timeframe.

### Outcome measures

The co-primary efficacy endpoints of the study will be MRI assessment of volume changes in knee cartilage from baseline to month 24, as well as the Western Ontario and McMaster Universities Index of OA (WOMAC) score [31] (Table 1). The secondary endpoints will be other knee structural changes (cartilage defects, tibial plateau bone area, and bone marrow lesions, meniscal tear and extrusion) from baseline to month 24, and lower limb muscle strength at months 3, 6, 12 and 24 (Table 1). 

**Table 1:** Timetable and measures to be made

	**Screening**	**Month(s)**				
		**0**	**3**	**6**	**12**	**24**
**Co-primary outcome measure**						
MRI (cartilage volume changes)		✓				✓
WOMAC		✓	✓	✓	✓	✓
**Secondary outcome measure**						
MRI (other structural changes)		✓				✓
Lower limb muscle strength		✓	✓	✓	✓	✓
**Other measures**						
Core musculature measure		✓			✓	✓
Hand grip strength		✓	✓	✓	✓	✓
Central and upper arm blood pressure		✓		✓	✓	✓
Aortic stiffness		✓		✓	✓	✓
Physical activity (IPAQ)		✓				✓
Body fat		✓			✓	✓
Low foot pain		✓	✓	✓	✓	✓
Low back pain		✓		✓	✓	✓
Depression		✓	✓	✓	✓	✓
Quality of life		✓		✓	✓	✓
Previous knee injury and occupation		✓				✓
Weight		✓	✓	✓	✓	✓
Height		✓				✓
Girth measurements		✓			✓	✓
Knee radiograph	✓					
Serum 25-(OH)D	✓		✓			✓
Serum calcium, phosphate, creatinine	✓		✓			
Sun exposure		✓		✓	✓	✓
Cigarette smoking		✓				✓
Diet (FFQ) and pedometer		✓				✓
Medications	✓	✓	✓	✓	✓	✓
Pill counts and adverse events		✓	✓	✓	✓	✓

Participants who withdraw within one year will be asked to have MRI at month 12; patients who withdraw after one year will be asked to have MRI straight away. MRI: magnetic resonance imaging; WOMAC: Western Ontario McMaster Universities Osteoarthritis Index; 25-(OHD)D: 25-hydroxy-vitamin D; FFQ: food frequency questionnaire.

#### *MRI assessment of knee structural changes*

Knees will be imaged in the sagittal plane on a 1.5-T whole body MRI unit using a commercial transmit-receive extremity coil. Fat-saturated T1-weighted spoiled gradient echo (GRE) and T2-weighted/proton density-weighted fast spin echo (FSE) sequences will be used. The images will be assessed by two readers blinded to the treatment according to the methods described in our previous publications [15,32].

Cartilage volume: the volumes of individual cartilage plates (medial tibial, lateral tibial and patella) are isolated from the total volume by manually drawing disarticulation contours around the cartilage boundaries on a section-by-section basis. Sagittal images will be obtained at a partition thickness of 1.5 mm and an in-plane resolution of 0.31 × 0.31 mm (512 × 512 pixels), then resampled by means of bilinear and cubic interpolation (area of 312 μm × 312 μm and multiplied by 1.5 mm thickness, continuous sections) for the final 3D rendering. Particular cartilage volume was then determined by summing all the pertinent voxels within the resultant binary volume.

Cartilage defects assessment: the cartilage defects (0 to 4) will be graded at medial tibial and femoral, lateral tibial and femoral, and patellar sites: grade 0, normal cartilage; grade 1, focal blistering and intracartilaginous low-signal intensity area with an intact surface and bottom; grade 2, irregularities on the surface or bottom and loss of thickness of less than 50%; grade 3, deep ulceration with loss of thickness of more than 50%; grade 4, full-thickness chondral wear with exposure of subchondral bone.

Knee tibial plateau bone area: the area of the medial and lateral tibial plateau bone will be measured manually on the three reformatted images closest to the tibial cartilage. An average of these three areas will be used as an estimate of the tibial plateau bone area.

Subchondral bone marrow lesions: This will be assessed on the T2-weighted MRI and defined as discrete areas of increased signal adjacent to the subcortical bone at the lateral, medial femur and/or tibia. Each bone marrow lesion will be scored on the basis of lesion size, for example, a lesion is scored as grade 1 if it occupies < 25% of the region; grade 2 if it occupies 25% to 50% of the region; or grade 3 if it occupies > 50% of the region.

Meniscal tear assessment: the menisci will be assessed in the sagittal view and confirmed in the coronal and axial views as previously described [33]. In brief, the presence or absence of a tear is based on the presence of a signal, which is line shaped, brighter than the dark meniscus, and reaches the surface of the meniscus at both ends within six defined regions (anterior horn, body, and posterior horn at both medial and lateral tibiofemoral compartments).

Mensical extrusion assessment: the extent of meniscal extrusion on the medial or lateral edges of the tibial femoral joint space for the anterior, body, and posterior horns of the menisci will be graded, where a score of 0 = no extrusion, 1 = partial meniscal extrusion, and 2 = complete meniscal extrusion with no contact with the joint space.

#### *Lower limb muscle strength*

This will be measured by dynamometry (TTM Muscle Meter, Tokyo, Japan) at the lower limb (involving both legs simultaneously). The muscles measured with this technique are mainly the quadriceps and hip flexors. The device will be calibrated by suspending known weights at regular intervals.

#### *WOMAC*

Knee pain will be assessed by both WOMAC pain subscale (walking on a flat surface, going up/down stairs, at night in the bed , sitting/lying and standing upright) and a 100 mm VAS.

#### *Other measurements*

Core musculature measure: Core muscle images will be taken at baseline and 12 months. Images of the core muscles (TrAb, internal oblique muscles and LM) are taken with real-time dynamic ultrasound using a fully featured big box diagnostic ultrasound machine (Phillips HDI 5000, Bothell, WA, US) with a hand held 7.5 mHz linear array transducer. Images are taken of right and left sides, both at rest and during contraction (drawing in of abdomen) using previously published protocols [34,35]. These measures have a high degree of reliability with an interclass correlation coefficient (ICC) > 0.90 across a range of studies [36].

Upper arm blood pressure, central blood pressure and aortic stiffness: Clinical upper arm blood pressure will be measured twice after 5 minutes seated rest using a validated device (Omron HEM-907, Kyoto, Japan). Seated clinical central blood pressure will be recorded (immediately after upper arm blood pressure) using radial applanation tonometry (SphygmoCor 8.1, AtCor Medical, Sydney, Australia). Aortic stiffness will be measured by electrocardiogram-gated, sequential carotid to femoral pulse wave velocity as per expert consensus [37].

Physical activity: Physical activity will primarily be assessed using a pedometer (SW 200 Digi-Walker, Yamax Corporation, Tokyo, Japan), which measures vertical displacement (steps per day). The pedometer will be worn for seven consecutive days on two occasions (baseline and 2 years) as up to seven days is required to accurately assess habitual physical activity [38]. We will also measure physical activity using the International Physical Activity Questionnaire (IPAQ) short version [39].

Body fat: Body fat will be assessed using bioelectrical impedance analysis (BIA) (BIA analyser, Quantum II, RJL Systems, Michigan, USA). Fat-free mass, % fat-free mass, fat mass and % fat mass will be assessed [40].

Hand grip strength: Hand grip strength will be assessed to the nearest kg in both the right and left hand using a hydraulic hand dynamometer (Saehan Corporation, Masan, Korea). Both hands will be alternately measured in triplicate.

Radiographic OA: this will be assessed at baseline by a standing semiflexed anterior-posterior (AP) radiograph as per the Altman atlas [29]. Radiographs will also be assessed simultaneously by two observers using the Osteoarthritis Research Society International (OARSI) atlas to score osteophytes and joint space narrowing on a four-point scale (0 to 3).

Laboratory measurements: serum 25-(OH)D will be assayed at month 0, 3 and 24, utilizing a Liquid Phase radioimmunoassay (Immunodiagnostics Systems Ltd, Boldon, Tyne & Wear, UK). Serum calcium, phosphate and renal function will be assessed at month 0 and 3 using routine biochemical methods.

Anthropometrics and other questionnaires: Height will be measured to the nearest 0.1 cm (with shoes removed) using a stadiometer (Leicester Height Measure, Invicta Plastics Ltd, Leicester, UK). Weight will be measured to the nearest 0.1 kg (with shoes and bulky clothing removed) using electronic scales (Heine S-7307, Heine, New Hampshire, USA). Waist and hip measurements will be assessed using a tape measure to the nearest 0.1 cm (Figure Finder Tape Measure, Novel Products Inc, Illinois, USA). Sun exposure, employment status and occupation, depression, smoking status, previous knee injury, dietary intake, low back and foot pain and quality of life will be assessed by questionnaires.

### Safety assessments

Spontaneously reported adverse events will be recorded throughout the study. Intensity and relationship with the study medication will be ascribed.

### Sample size

All sample size calculations assume α = 0.05 and β = 0.20 and are performed based upon formulae provided by Cohen [41]. Table 2 describes the sample size (each arm) needed to detect the specified differences between the placebo and vitamin D arms with at least 80% power for each outcome. 

**Table 2:** Sample size calculation

	**Mean (SD)**	**Detectable difference**	**Calculated sample size (per arm)**
Loss of volume of medial tibial cartilage	4.5% ± 6.5%	2.16%	143
Increase in medial tibial bone area	1.6% ± 2.8%	0.9%	153
Incidence of knee cartilage defects	80%	15%	136

Previous studies, including our own, suggest that OA patients have a loss of cartilage volume of 4 to 5% per year at different joint sites, respectively [42]. Vitamin D supplementation in doses ranging from 400 to 800 IU/d increased the serum level of 25-(OH)D by 27 nmol/liter per year in 7,964 men and women from five studies [43]. We estimate from our published data [15] that this change will lead to absolute reduction in loss of cartilage volume by 2.2% at the medial tibial site after vitamin D supplementation. The sample size that is needed to detect this difference is calculated (Table 2).

We have shown that male OA patients have an increase in medial tibial bone area of 1.6 ± 2.8% per year [44], and an incidence of knee cartilage defects of 80% over 2 years [45]. There are no data known to the investigators about the associations between change in vitamin D and change in bone area or cartilage defects. However, healthy subjects have been shown to have an increase in tibial bone area of 0.7% per year (CD *et al*., unpublished), and an incidence of knee cartilage defects of 65% over 2 years in older people [46]. Assuming that changes in cartilage defects and bone area in OA patients will be suppressed by vitamin D supplementation to the levels in the healthy subjects, the sample size needed to detect these differences is given in Table 2.

Therefore, 200 patients in each arm (allowing for a 20% dropout over the trial) will be sufficient to detect the differences between treatment groups.

### Analysis plan

Statistical primary comparisons for total and subscale WOMAC scores will be made using a repeated measures mixed model with terms for treatment, month, center and the corresponding baseline values as the covariates. The independent *t*-tests will be used to compare changes between groups in quantitative data from baseline to the end of follow-up. Linear regression (annual changes in cartilage volume, cartilage defects, bone area and muscle strength as the dependent variables, and treatment as the independent variable) and logistic regression (development/progression of bone marrow lesions and meniscal abnormalities as the dependent variables, treatment as the independent variable) analyses will be applied in univariate and multivariate modeling adjusted for age, sex, body mass index, baseline 25-(OH)D and other disease status.

In secondary analysis of loss in cartilage volume, the minimal clinically important differences (MCID) in cartilage volume will be calculated [47] and logistic regression will be used to determine the association between cartilage loss (> = MCID vs. < MCID) and treatment before and after adjustment for the covariates described above.

Both intention to treat and per protocol analyses will be utilized. Per protocol will be defined as achieving a 25-(OH)D level > 60 nmol/liter at month 3. The last observation carried forward method will be used in the analysis of all outcomes among patients who made at least one follow-up visit but did not complete the whole study.

### Data integrity and management

All data obtained will be kept strictly confidential and will be stored electronically on a database with secured and restricted access. Data transfer will be encrypted and any information capable of identifying individuals will be removed.

### Withdrawal

If a participant withdraws or is removed from the study, the reason and date of discontinuation will be recorded. Any participant who withdraws within year 1 will be asked to have MRI at the end of year 1; participants withdrawing after year 1 will be asked to have MRI on leaving the study.

### Monitoring

The trial will be overseen and monitored by a project manager. The project manager will visit each site to examine trial procedures to ensure data quality and compliance with the trial protocol.

## Discussion

We have proposed this protocol to determine if vitamin D supplementation can slow disease progression in patients with knee OA. Vitamin D may have beneficial effects for the treatment of OA, although there are currently no recommended guidelines for this approach [48]. Hence, well-designed randomized controlled trials are required to test if vitamin D has disease-modifying and pain-relieving effects. Such studies also need an appropriate follow-up period to capture joint structural changes using objective measurements over the course of OA, and this has been incorporated into the design of the VIDEO study.

Assessing disease-modifying effects on OA requires an accurate measurement tool that is able to evaluate improvements in cartilage and joint health. Radiographic assessment of OA is two-dimensional, lacks sensitivity for changes over a short period and is highly susceptible to measurement error through factors such as variation in joint positioning [49]. MRI allows direct, accurate and reliable assessment of joint structural changes over time. These structural changes include cartilage loss, cartilage defects, increased tibial bone area, subchondral bone marrow lesions, meniscal tears and meniscal extrusion. Almost all these structural changes are predictive of total knee replacement [45,50,51], suggesting they are clinically relevant. Simultaneously, we will assess change in knee pain over time using WOMAC as a co-primary endpoint. Thus, the findings from this study will show whether vitamin D supplementation has both disease-modifying and symptom-relieving effects.

As suggested by the 2011 Endocrine Society Clinical Practice Guideline, all adults aged 50 to 70 years, and those over 70 years old require at least 600 and 800 IU/d of vitamin D respectively, to maximize bone health and muscle function. To raise blood levels of 25-(OH)D above 75 nmol/liter (the lowest sufficient threshold) requires at least 1500 to 2000 IU/d of supplemental vitamin D [52]. Thus, our study design provides a dose of 50,000 IU monthly to achieve serum 25-(OH)D levels above 60 nmol/liter in all compliant subjects [53]. This method will be less costly and will be more convenient than daily treatment. Toxicity is extremely unlikely with this dose.

Two sub-studies will simultaneously be included in this trial. Firstly, we will examine the effects of vitamin D on the function of the deep lumbo-pelvic stabilizing muscles. Besides implications for low back pain, in healthy people core muscles have also been implicated in varying aspects of physical function. The lateral abdominal muscles are theorized to control movement and provide stability to the trunk for functional activities and this is supported in a number of studies [54]. Vitamin D supplementation may have beneficial effects on functionally important core muscles. Secondly, we will determine the effect of vitamin D supplementation on blood pressure and aortic stiffness. A recent systematic review suggested that there is accumulating evidence to support the hypothesis that vitamin D deficiency contributes to hypertension, and randomized controlled trials (RCTs) are greatly needed to clarify and to definitively prove the effect of vitamin D on blood pressure [55]. The VIDEO study aims to be the first to assess this.

In summary, knee OA is a major, but poorly understood, public health problem. Vitamin D deficiency may play a role in the progression of OA, and based on our novel preliminary data, the VIDEO study has been designed to determine whether intervening with vitamin D supplementation can in fact slow the progression of this disease and relieve knee pain. If correcting vitamin D deficiency can reduce rates of cartilage loss to lower levels as seen in older people without OA, it will significantly prolong the time it takes to reach end-stage OA eventually requiring joint replacement. This suggests great potential for substantial cost savings through reductions in joint replacement surgery, as well as potential for great improvements in the quality of life for people with OA. The success of this study will provide scientific evidence for using a cost-effective and innovative approach to addressing this clinically significant problem and will lend itself to an easy public health intervention.

## Trial status

Upon submission, VIDEO study is in the process of patient recruitment.

## Abbreviations

25-(OH)D: 25-hydroxy-vitamin D; ACR: American College of Rheumatology; AQOL: assessment of quality of life; BIA: bioelectrical impedance analysis; CRF: case report form; FSEL: fast spin echo; FFQ: food frequency questionnaire; GRE: gradient echo; ICC: interclass correlation coefficient; IPAQ: International Physical Activity Questionnaire; LM: lumbar multifidus; MCID: minimal clinically important differences; MMP: metalloproteinase; MRI: magnetic resonance imaging; OA: osteoarthritis; OARSI: Osteoarthritis Research Society International; PHQ-9: Personal Health Questionnaire Depression Scale-9; PGE_2_: prostaglandin E_2_; RAAS: renin-angiotensin-aldosterone system; RCT: randomized controlled trial; SOP, standard operating procedure; TASOAC, Tasmania Older Adult Cohort; TrAb: transversus abdominus; VAS: visual analogue scales; VIDEO: Vitamin D Effects on Osteoarthritis; VDR: vitamin D receptors; WOMAC: Western Ontario McMaster Universities Osteoarthritis Index.

## Competing interests

The authors declare that they have no competing interests.

## Authors’ contributions

CD and GJ conceived the study, CD, GJ, FC, TW, AW, JS, KN and YC participated in its design and coordination, and performed the research. YC, GJ, KN and CD drafted the manuscript. All authors revised the manuscript and gave final approval of the version to be submitted.

## Funding

The VIDEO study is supported by the National Health & Medical Research Council (NHMRC ID 605501).
